# Pharmacist‐Led Intervention on the Inappropriate Use of Stress Ulcer Prophylaxis Pharmacotherapy in Intensive Care Units: A Systematic review

**DOI:** 10.3389/fphar.2021.741724

**Published:** 2021-10-25

**Authors:** Peipei Xu, Qiusha Yi, Cuitong Wang, Linan Zeng, Keith M. Olsen, Rongsheng Zhao, Mingyan Jiang, Ting Xu, Lingli Zhang

**Affiliations:** ^1^ Department of Pharmacy/Evidence-Based Pharmacy Center, West China Second University Hospital, Sichuan University, Chengdu, China; ^2^ Key Laboratory of Birth Defects and Related Diseases of Women and Children (Sichuan University), Ministry of Education, Chengdu, China; ^3^ West China School of Medicine, Sichuan University, Chengdu, China; ^4^ West China School of Pharmacy, Sichuan University, Chengdu, China; ^5^ Department of Pharmacy Practice and Science, College of Pharmacy, University of Nebraska Medical Center, Omaha, NE, United States; ^6^ Department of Pharmacy, Peking University Third Hospital, Beijing, China; ^7^ Department of Pharmacy, the First Hospital of China Medical University, Shenyang, China; ^8^ Department of Pharmacy, West China Hospital, Sichuan University, Chengdu, China

**Keywords:** pharmacist-led, stress ulcer prophylaxis, intensive care unit, systematic review, quality improvement

## Abstract

**Background:** Pharmacist’s direct intervention or participation in multidisciplinary management teams can improve the clinical outcome and quality of life of patients. We aimed to determine the effectiveness of pharmacist-led interventions on the inappropriate use of stress ulcer prophylaxis (SUP) pharmacotherapy in intensive care units (ICUs).

**Methods:** A systematic review was performed for relevant studies using searched PubMed, EMBASE (Ovid), the Cochrane Library, Cochrane Central Register of Controlled Trials (CENTRAL), and four Chinese databases from the establishment of databases to 12 March 2020. We conducted a descriptive analysis of participants, the intervention content and delivery, and the effects on inappropriate medication rates.

**Results:** From 529 records, 8 studies from 9 articles were included in the systematic review. The time of appropriateness judgment and the criteria of “appropriate” varied from included studies. Pharmacist interventions mainly included clarifying indications for SUP pharmacotherapy, education and awareness campaign, reviewed patients on SUP pharmacotherapy during rounds, and adjustments of drug use. Five (62.5%) studies found a significant intervention effect during hospitalization, while 2 (25%) studies at ICU transfer and 2 (25%) studies at hospital discharge. 4 (50%) studies identified the complications related to SUP pharmacotherapy and found no significant difference. 4 (50%) studies declared the pharmacist-led interventions were associated with cost savings.

**Conclusion:** Pharmacist-led intervention is associated with a decrease in inappropriate use of SUP pharmacotherapy during hospitalization, at ICU transferred and hospital discharged, and a lot of medical cost savings. Further research is needed to determine whether pharmacist-led intervention is cost-effective.

## Introduction

With the advancement of pharmacy directed patient care, the role of pharmacists has expanded from the traditional task of distributing medications and providing basic drug information to a team-based clinical role providing patient-centered medication therapy management (Albanese et al., 2010). Many studies have confirmed that pharmacist’s direct intervention or participation in multidisciplinary management teams can improve the clinical outcome and quality of life of patients by optimizing the use of drugs in different disease processes (Thomas et al., 2014; Dixon et al., 2016; Greer et al., 2016; van Eikenhorst et al., 2017; De Barra et al., 2018; McNab et al., 2018; Mes et al., 2018; Alshehri et al., 2020).

As a member of a multidisciplinary management team, pharmacists make full use of their professional knowledge and clinical experience to perform an important role in the care of intensive care unit (ICU) patients (Preslaski et al., 2013). A previous systematic review sufficiently dissected the impact on patient outcomes of pharmacist participation in multidisciplinary critical care teams (Lee et al., 2019). This paper clarified pharmacist’s participation improved patient outcomes including mortality, ICU length of stay in mixed ICUs, and preventable/nonpreventable adverse drug events (Lee et al., 2019).

Patients admitted to the intensive care unit (ICU) have a risk of stress-related mucosal damage (SRMD) that may evolve into ulcers and hemorrhage (Marik et al., 2010). SRMD is apparent in 75–100% of critically ill patients within 24 h after admission to an ICU(Metz, 2000; Fennerty, 2002). And the prevalence of gastrointestinal bleeding (GIB) ranges from 5.6 to 9.0% in recent reports (Selvanderan et al., 2016; Alhazzani et al., 2017; Krag et al., 2018) and has been associated with an increased risk of death and ICU length of stay (Krag et al., 2015). Preventing potential progression from SRMD to GI bleeding, acid suppression therapies (AST) are often overused for stress ulcer prophylaxis (SUP) (Farrell et al., 2010; Frandah et al., 2014; Buckley et al., 2015; Hammond et al., 2017; Masood et al., 2018). Inconsistent recommendations on the initiation of SUP in existing guidelines, including mechanical ventilation, chronic liver disease, coagulopathy, head injury, thermal injury, and multiple trauma, *etc* (Therapeutic Guidelin, 1999; Madsen et al., 2014)^)^ *.* Previous studies on the prescription behaviors showed that approximate 75% of the patients received SUP during ICU stay, 14.4–42% of whom had no identifiable risk of stress ulcer (Farrell et al., 2010; Frandah et al., 2014; Buckley et al., 2015; Hammond et al., 2017; Masood et al., 2018). Although SUP has been proved effective in decreasing the incidence of gastrointestinal bleeding (Krag et al., 2014; Barbateskovic et al., 2019), it also leads to increased myocardial ischemia, Clostridium (C.) difficile infection, hospital-acquired pneumonia, increased hospitalization and prescription costs (Driks et al., 1987; Heidelbaugh and Inadomi, 2006; Grube and May, 2007; Lin et al., 2010; Marik et al., 2010; Alhazzani et al., 2013). The overuse of SUP may lead to increased adverse events, drug-drug interactions, and increased hospital and prescription costs.

Although several studies had examined the impact of pharmacist-led de-escalating SUP pharmacotherapy, they had not been reviewed. Our systematic review aimed to determine the effectiveness of pharmacist-led interventions on the inappropriate use of SUP pharmacotherapy in ICUs.

## Methods

This systematic review conformed to the PRISMA statement and Synthesis without meta-analysis (SWiM) reporting guideline and was registered on PROSPERO (CRD42021239821) (Liberati et al., 2009; Campbell et al., 2020).

### Eligibility Criteria

We included studies evaluating the impact of pharmacist-led interventions on the use of stress ulcer prophylaxis in patients or in the intensive care unit. We included randomized controlled trials (RCTs), cohort studies, and case-control studies. There were no restrictions on language and publication time.

Inclusion criteria followed the Participant-Intervention-Comparison-Outcome-Study Design (PICOS) framework (Higgins, 2011). Participants were patients in intensive care units who were critically ill or a short stay for observation. We excluded studies that focused on all departments but did not separately provide data from ICU departments. The intervention content could be provided in part or whole by the pharmacist (i.e., the pharmacist-led). The interprofessional approaches were included only when pharmacists as part of a shared-care approach and as the primary decision makers. We included studies of any design with a comparator group of usual care or other healthcare’s intervention. We included studies with the incidence pharmacotherapeutic intervention in SUP as a primary or secondary outcome. We did not limit the observation time of outcome indicators, whenever during hospitalization, at ICU discharge, or hospital discharge.

### Search and Information Sources

We searched Chinese Biomedical Literature (Chinese), Cochrane Central Register of Controlled Trials (English), the Cochrane Library (English), China National Knowledge Infrastructure (Chinese), EMBASE (Ovid, English), PubMed (English), VIP (Chinese) and Wanfang (Chinese) from the establishment of databases to March 12, 2020. We obtained additional articles by hand-searching reference lists of systematic reviews and other articles and from peer-reviewers.

Our search strategy used database-specific vocabulary (e.g., Medical Subject Headings) and free-text terms text expanding from “stress ulcer”, “pharmacist”, and “critically ill”. For “stress ulcer prophylaxis”, in addition to the original expanded vocabulary, we searched clinical symptoms (such as gastrointestinal bleeding and gastric mucosal lesion) and specific preventive drugs (including H-2 receptor antagonist, proton pump inhibitors, and sucralfate).

The search strategy was developed specifically for each database (Supplementary Table S1).

### Study Selection

We used EndNote (version X8) reference manager for records management and duplicates removal. Two investigators (WCT and XPP) screened all titles and abstracts. Once relevant articles were screened in, two investigators (WCT and XPP) independently screened full-text articles. All inconsistent inclusion decisions were resolved through consensus with a third reviewer (YQS).

### Data Collection and Quality Assessment

Study data were extracted by one investigator (WCT) using specifically developed data extraction forms and checked by another investigator (XPP). Extracted data contained: (Albanese et al., 2010): author’s name, year, the country of study origin and study purpose; (De Barra et al., 2018); method (study design and information of study quality according to quality assessment criteria of different types of studies); (Mes et al., 2018); participant and setting (sample size, age, inclusion and exclusion criteria, indications for the use and cessation of SUP pharmacotherapy, the definition of rational use, and setting); (Greer et al., 2016); intervention (composition, implementer, and formation method); (Alshehri et al., 2020); outcomes (the incidence of the inappropriate use of SUP pharmacotherapy, cost of medications used for SUP, and complications of SUP pharmacotherapy; and (McNab et al., 2018) confirmation of eligibility for review.

We used the Newcastle-Ottawa Scale for assessing the risk of bias of cohort studies (Peterson et al., 2011).

### Data Synthesis and Analysis

The primary outcome was the incidence of inappropriate use of SUP pharmacotherapy. Secondary outcomes included complications related to SUP pharmacotherapy and economic outcomes.

As the heterogeneity of the research inclusion criteria, the denominator was inconsistent when calculating the inappropriate rate. Some studies use all patients in the ICU as the denominator, while others use the patients receiving SUP pharmacotherapy during ICU hospitalization. Therefore, we recalculated the rate using the SUP pharmacotherapy population during ICU hospitalization as the denominator to get the standardized metric. We excluded patients with chronic AST prior to admission if there was no reconsideration of the appropriateness of chronic AST.

Due to the expected heterogeneity of participants, interventions, and the definition of inappropriate, it was hard to group studies for synthesis and undertake a meta-analysis. Instead, we conducted a descriptive analysis of participants, the intervention content and delivery, and the effects on inappropriate medication rates. And as recommended by the Cochrane handbook for systematic reviews of interventions, we used vote counting, in which the number of favorable studies is counted and compared with the number of unfavorable studies (Cumpston et al., 2019). Specifically, studies were assessed according to whether or not they found statistically significant evidence supporting the appropriate use of SUP pharmacotherapy: effectiveness (the inappropriate rate at initiation, ICU transfer and hospital discharge), safety (not increase the incidence of complications), and economy. The balance of positive vs. negative studies was used to determine the answer to the review questions.

As we we adjusted the denominator to recalculate the rate, we didn’t rely on *p*-values reported by the authors of the primary studies. Chi-square tests were used for categorical group comparisons based on pre- and post-intervention groups. Data were analyzed using IBM SPSS Statistics for Windows v22.0 (IBMCorp., Armonk, NY). *p*-values<0.05 were considered statistically significant. For economic outcomes, we unified the monetary unit to the United States dollar (1 Australischer Dollar = 0.778 US Dollar; 1 Canadian dollar = 0.7891 United States Dollar).

## Results

### Study Selection

**FIGURE 1:**
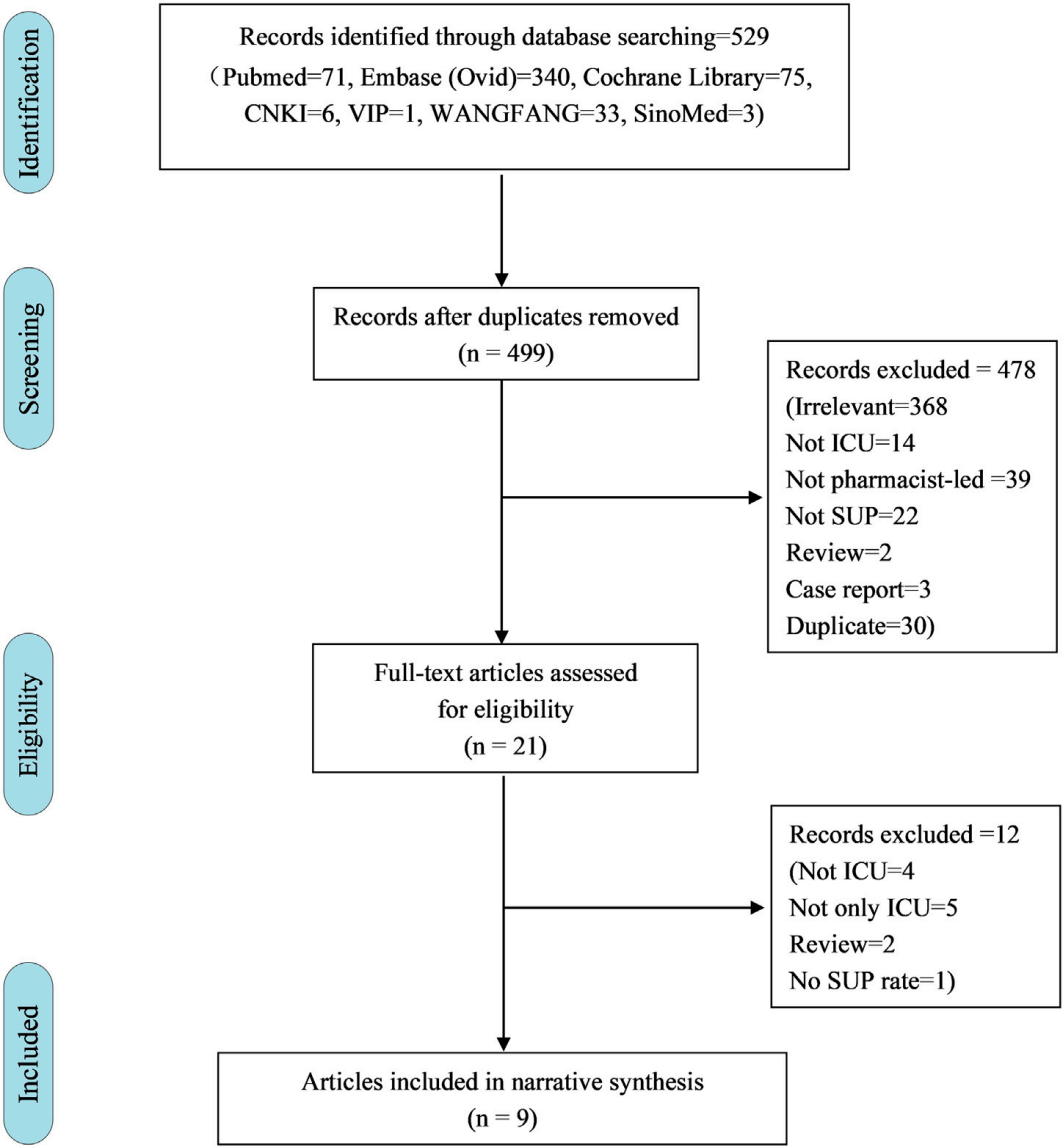
Flow chart for screened articles.

A total of 529 studies were retrieved from the databases. From the total, 478 studies were excluded based on titles and abstracts and 12 studies were excluded based on full-text articles (Figure 1). Primary reasons for exclusion were non-ICU, non-pharmacist-led intervention, non-SUP-related medications, cannot extract ICU data separately, reviews, case reports, and duplicate literature (Figure 1). We included 8 studies from 9 articles in the narrative synthesis (Coursol and Sanzari, 2005; Wohlt et al., 2007; Hatch et al., 2010; Tasaka et al., 2014; Buckley et al., 2015; Guobin et al., 2015; Hammond et al., 2017; Masood et al., 2018; Anstey et al., 2019). All studies were cohort studies, of which 6 (75.0%) were retrospective and the other 2 (25.0%) were prospective. Observation periods ranged from 2 weeks to 6 months. All studies assessed appropriateness during ICU hospitalization. In addition, 4 (50.0%) studies assessed appropriateness at ICU transfer and hospital discharge at the same time (Table 1).

**TABLE 1:** Characteristics of included studies.

Study ID	Country	Study design	Center	Sample size		Observation periods (months)	Outcome measurement time point	Significant intervention effect?
				Pre-	Post-			
Anstey et al. (2019)	Australia	Prospective cohort study	5	531	393	5	ICU hospitalization	No
							Hospital discharge	Yes
Masood et al. (2018)	United States	Retrospective cohort study	1	162	202	1	ICU hospitalization	Yes
Hammond et al. (2017)	United States	Retrospective cohort study	1	101	118	6	ICU hospitalization	Yes
							ICU transfer	No
							Hospital discharge	No
Buckley et al. (2015)	United States	Retrospective cohort study	1	174	167	1	ICU hospitalization	Yes
							ICU transfer	Yes
							Hospital discharge	Yes
Guobin et al. (2015)	China	Retrospective cohort study	1	20	20	1	ICU hospitalization	No
Tasaka et al. (2014)	United States	Retrospective cohort study	1	75	56	0.5	ICU hospitalization	Yes
							ICU transfer	No
							Hospital discharge	No
Wohlt et al. (2007) (pre-)	United States	Retrospective cohort study	1	494	458	1	ICU transfer	Yes
Hatch et al. (2010) (post-)								
							Hospital discharge	Yes
Coursol and Sanzari (2005)	Canada	Prospective cohort study	1	303	252	1	ICU hospitalization	Yes

### Participant Characteristics

Most studies included adult patients (6, 75.0%) and the other 2 (25.0%) did not specify the study population (Table 2). Regarding the type of ICU, 2 (25.0%) studies included patients in medical and surgical ICUs, 2 (25.0%) studies only included patients in medical ICU, and the other 4 (50.0%) studies did not specify the ICU category. 5 (62.5%) studies included all patients admitted to the ICU, while 3 (37.5%) studies only focused on patients who received AST. Inclusion criteria varied between studies but most of them (5, 62.5%) excluded patients having an additional indication for AST (e.g., active GIB, active peptic ulcer disease, and Zöllinger-Ellison syndrome) or they were not indicated for SUP pharmacotherapy regardless of risk factors (e.g., total gastrectomy) (Coursol and Sanzari, 2005; Hatch et al., 2010; Tasaka et al., 2014; Buckley et al., 2015; Hammond et al., 2017).

**TABLE 2:** Participant characteristics of included studies.*^△^

Study ID	Age (years)	Male sex	Department	Inclusion criteria	Exclusion criteria
Anstey et al. (2019)	T: 59 (40–71)	T: 230 (58.5%)	ICU	All adult (≥18 years)	Patients aged<18 years·
	C: 60 (42–71)^a^	C: 301 (56.7%)		hospitalized patients	Cases with missing AST data
Masood et al. (2018)	NR	NR	Medical ICU	·All patients admitted to the ICU	Patients had acute GI bleeding
Hammond et al. (2017)	T: 56.24±18.35	NR	Medical ICU	·All adult (≥18 years) hospitalized	Patients possessed a current diagnosis of GIB
	C: 51.07±4.52			patients·Patients with an order for AST	Patients on AST prior to admission to the ICU
					Patients with a history of Zöllinger-Ellison syndrome
Buckley et al. (2015)	T: 55.5±18.8 C: 58.3±17.1	T: 110 (65.9%) C: 90 (51.7%)	ICU	·All adult (≥18 years) hospitalized patients Patients received either an H2RA or PPI	Patients had GI diseases Patients receiving AST prior to admission to the ICU
Guobin et al. (2015)	NR	NR	ICU	·All patients admitted to the ICU	—
				Patients with an order for AST	
Tasaka et al. (2014)	≥18	NR	Medical and surgical ICU	·All adult (≥18 years) hospitalized patients	Patients had: Active GIB Active peptic ulcer disease Total gastrectomy Solid organ transplant Dual antiplatelet therapy Concurrent antiplatelet and anticoagulation therapy Nonenteric coated pancrelipase *via* gastric feeding tube
Wohlt et al. (2007) **(pre-)** Hatch et al. (2010) **(post-)**	T: 55±19 C: 54±19	T: 269 (58.7%) C: 287 (58.1%)	Medical and surgical ICU	All adult (≥18 years) hospitalized patients	Patients had a current diagnosis of gastrointestinal bleeding, Zöllinger-Ellison syndrome, prisoner status Patients died while in the hospital
Coursol and Sanzari (2005)	18–90	T: 157 (62.3%) C: 191 (63.0%)	ICU	All adult (≥18 years) hospitalized patients	Patients refused treatment Patients died <24 h after admission Patients who pregnant Patients with gastrointestinal bleeding, or an active ulcer, or Zöllinger–Ellison syndrome

a T: post-intervention group; C: pre-intervention group.

b NR: not reported.

### Risk of Bias in the Included Studies

The NOS quality stars ranged between 5 and 7, and the average score was 5.88 for cohort studies (Table 3). Six studies had an overall fair quality, which indicated a low risk of bias. Two studies were determined as poor quality, indicating the risk of bias. 7 (87.5%) studie’s exposed cohort were from single center which is not representative. All studies had no quantitative description of exposure, which means the exposure was uncertain.

**TABLE 3:** Risk of bias of included studies

		Anstey et al. (2019)	Masood et al., 2018	Hammond et al. (2017)	Buckley et al. (2015)	Tasaka et al. (2014)	Guobin et al. (2015)	Wohlt et al. (2007) (pre-)	Hatch et al. (2010) (post-)	Coursol and Sanzari (2005)
**SELECTION**	Representativeness of the Exposed Cohort	★	0	0	0	0	0	—	0	0
	Selection of the Non-Exposed Cohort	★	★	★	★	★	★	—	★	★
	Ascertainment of Exposure	0	0	0	0	0	0	—	0	0
	Demonstration That Outcome of Interest Was Not Present at Start of Study	★	★	★	★	★	★	—	★	★
**COMPARABILITY**	Comparability of Cohorts on the Basis of the Design or Analysis	★	0	★	★	0	★	—	★	★
**OUTCOME**	Assessment of Outcome	★	★	★	★	★	★	—	★	★
	Was Follow-Up Long Enough for Outcomes to Occur	★	★	★	★	★	★	—	★	★
	Adequacy of Follow Up of Cohorts	★	★	★	★	★	★	—	★	★
**TOTAL**	—	7	5	6	6	5	6	—	6	6
**OVERALL QUALITY**	—	**Fair**	**Poor**	**Fair**	**Fair**	**Poor**	**Fair**	—	**Fair**	**Fair**

### Intervention Content and Delivery

Pharmacist interventions mainly included 4 aspects: 1) clarify indications for SUP pharmacotherapy; 2) education and awareness campaign; 3) reviewed patients on SUP pharmacotherapy during rounds; 4) adjustments of drug use (Table 4).

**TABLE 4:** Intervention content and delivery of included studies

Intervention									Details	
Study ID	Indication			Education		Rounds	Adjustments of drug use	Design	Content	Primary implementor
	Local SUP guidelines/protocol	Algorithm		Medical staff	Materials					
Anstey et al. (2019)	**●**	—		—	—	—	**●**	NR	(a) A site-based dissemination of locally produced SUP prescription guidelines	NR
									(b) ICU pharmacist-led discontinuation of SUP prior to ICU discharge	Pharmacists
Masood et al. (2018)	—	—		**●**	**●**	**●**	**●** (prescribe authority)	NR	(a) Pharmacists reviewed patients on SUP during medical ICU rounds	Pharmacists
									(b) Pharmacists made appropriate changes (prescriptive authority) according to the guidelines	Pharmacists
									(c) Residents and fellows were educated and house staff were provided with printed copies of SUP indications	Pharmacists
Hammond et al. (2017)	—	—		**●**	**●**	**●**	—	NR	(a) A pharmacist provided medical residents and pulmonary/critical care fellows with an educational intervention	Pharmacists
									(b) Supplied a pocket card on SUP initiation and choice of agent	Multidisciplinary team
									(c) A pharmacist rounded with the medical ICU treatment team	Pharmacists
Buckley et al. (2015)	**●**	—		—	—	—	**●** (prescribe authority)	NR	(a) An institutional SUP prescription protocol	Pharmacists
									(b) Clinical pharmacists to initiate, modify, or discontinue stress ulcer prophylaxis	Pharmacists
Guobin et al. (2015)	—	—		—	—	—	—	NR	NR	pharmacists
Tasaka et al. (2014)	**●**	—		**●**	—	—	**●**	NR	(a) An institution SUP guideline	NR
									(b) An education and awareness campaign	NR
									(c) A pharmacist-led intervention	Pharmacists
Wohlt et al. (2007) (pre-)	—	—		—	**●**	**●**	**●**	NR	(a) A memorandum and a pocket card	Pharmacists
Hatch et al. (2010) (post-)									(b) Pharmacists also conducted medication reconciliation during daily patient care rounds and at discharge	Pharmacists
Coursol and Sanzari (2005)	—	**●**		—	—	—	—	NR	Stress Ulcer Prophylaxis Algorithm	pharmacists
**Amount**	**4**	—		—	**4**	**3**	**5**	—	—	—

4 (50%) studies clarified the indication for the initiation and discontinuation of SUP pharmacotherapy by developing locally SUP pharmacotherapy guidelines/protocol or algorithm (Coursol and Sanzari, 2005; Tasaka et al., 2014; Buckley et al., 2015; Anstey et al., 2019). 4 (50%) studies provided the medical staff with an educational intervention and/or supplied a pocket card of SUP pharmacotherapy indications for reference (Hatch et al., 2010; Tasaka et al., 2014; Hammond et al., 2017; Masood et al., 2018).

In 3 (37.5%) studies, pharmacists reviewed each patient on SUP pharmacotherapy during medical ICU rounds (Hatch et al., 2010; Hammond et al., 2017; Masood et al., 2018). In 5 (62.5%) studies, pharmacists made appropriate changes on SUP pharmacotherapy, in which 2 (25.0%) studies gave the pharmacist prescriptive authority to make such changes (i.e., initiate, continue, discontinue, or modify the route of medication administration) for SUP pharmacotherapy only (Hatch et al., 2010; Tasaka et al., 2014; Buckley et al., 2015; Masood et al., 2018; Anstey et al., 2019).

### Synthesis of Results

#### Effects on Inappropriate Use of SUP Pharmacotherapy

To clarify the definition of “inappropriate”, we first clarified the indication of SUP pharmacotherapy in all studies. Based on the most recent published guidelines and the latest evidence at the time of the study’s initiation, the indications for and cessation of SUP pharmacotherapy were different in each study (Supplementary Tables S2, 3). For the initiation of SUP pharmacotherapy, it involved 12 major risk factors (to meet one) and 14 minor risk factors (to meet two or more). The most common major risk factors were mechanical ventilation for >48 h and coagulopathy which were used by 7 (87.5%) studies. The common minor risk factors were high-dose glucocorticoid use and severe sepsis or septic shock which were used by 5 (62.5%) studies and 4 (50.0%) studies. For the cessation of SUP pharmacotherapy, 4 (50.0%) studies specified that SUP pharmacotherapy should be ceased when there is no ongoing indication (Tasaka et al., 2014; Buckley et al., 2015; Hammond et al., 2017; Anstey et al., 2019). 2 (25.0%) studies specified that SUP pharmacotherapy should be ceased when patients are discharged from ICU(Tasaka et al., 2014; Masood et al., 2018). 1 (12.5%) study specified that SUP pharmacotherapy should be ceased when patients received enteral feeding (Anstey et al., 2019). 3 (37.5%) studies did not specify the cessation of SUP pharmacotherapy (Coursol and Sanzari, 2005; Hatch et al., 2010; Guobin et al., 2015).

**FIGURE 2:**
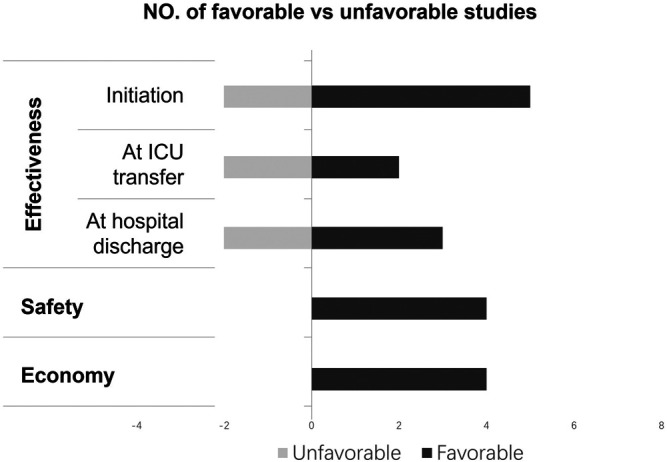
Synthesis of finding fromthe included studies (vote counting).

Between pre- and post-intervention groups, the assessment time of appropriateness varied from studies (Figure 2; Table 5). Seven studies comprised the incidence of inappropriate SUP initiation during ICU hospitalization, of which 5 (71.4%) studies found a significant intervention effect (Coursol and Sanzari, 2005; Tasaka et al., 2014; Buckley et al., 2015; Hammond et al., 2017; Masood et al., 2018). Four studies comprised the incidence of inappropriate continuation of SUP pharmacotherapy at ICU transfer, of which 2 (50.0%) studies found a significant intervention effect (Hatch et al., 2010; Buckley et al., 2015). Five studies included the incidence of inappropriate continuation of SUP pharmacotherapy at hospital discharge, of which 3 (60.0%) studies found a significant intervention effect (Hatch et al., 2010; Buckley et al., 2015; Anstey et al., 2019).

**TABLE 5:** The rate of inappropriate use of SUP pharmacotherapy.

Study ID	Rate of inappropriate use of SUP pharmacotherapy								
	Initiation of SUP			Continuation of SUP at ICU transfer			Continuation of SUP at hospital discharge		
	Pre-	Post-	*p*	Pre-	Post-	*p*	Pre-	Post-	*p*
Anstey et al. (2019)	19.81%	25.49%	0.198	—	—	—	36.79%	7.19%	<0.001
Masood et al. (2018) ^a^ ^b^	26.75%	7.14%	<0.001	—	—	—	—	—	—
Hammond et al. (2017)	23.76%	12.71%	0.033	60.40%	53.39%	0.297	17.82%	13.56%	0.385
Buckley et al. (2015)	14.38%^a^	6.03%^a^	<0.001	67.82%	38.92%	<0.001	29.89%	3.59%	<0.001
Guobin et al. (2015)	0.00%	0.00%	—	—	—	—	—	—	—
Tasaka et al. (2014)	21.26%^a^	9.09%^a^	0.004	8.00%	3.57%	0.498	6.67%	0.00%	0.131
Wohlt et al. (2007) (pre-) Hatch et al. (2010) (post-)	—	—	—	52.94%	27.27%	<0.001	26.89%	15.74%	0.003
Coursol and Sanzari (2005)	95.74%	88.24%	0.033	—	—	—	—	—	—

a The rate was calculated based on patient-day.

b Only one study (Masood 2018) included inappropriate use of SUP on patients who changed oral chronic AST use into intravenous route.

#### Effects on Complications and Economic Outcomes

Four studies identified the complications related to SUP pharmacotherapy (Figure 2; Table 6). There was no significant difference in the incidence of Clostridioides difficile-associated disease, pneumonia or hospital-acquired pneumonia, gastrointestinal bleeding, and thrombocytopenia between pre- and post-intervention groups.

**TABLE 6:** Complications related to SUP.

Study ID	Event	Pre-		Post-		*p*
		n	N	n	N	
Anstey et al. (2019) ^a^	*C. difficile*-associated disease	7	531	1	393	0.172
Hammond et al. (2017)	*C. difficile*	0	101	0	118	—
	Pneumonia	5	101	6	118	0.964
	Stress-related mucosal bleeding	1	101	0	118	0.938
Buckley et al. (2015)	Hospital-acquired pneumonia	29	174	25	167	0.668
	*C. difficile*-associated diarrhea	15	174	18	167	0.500
	Thrombocytopenia	11	174	5	167	0.146
	Gastrointestinal bleed	8	174	4	167	0.270
Coursol and Sanzari (2005) ^a^	Significant bleeding	2	303	3	252	0.836

a The incident is based on all ICU populations, not just SUP populations.

4 (50%) studies explored the economic benefits of pharmacist-led interventions improving SUP pharmacotherapy (Figure 2; Table 7) (Coursol and Sanzari, 2005; Buckley et al., 2015; Masood et al., 2018; Anstey et al., 2019). Anstey 2019 determined the extrapolated direct savings to all Australian intensive care units from reduced SUP pharmacotherapy were $1.61 million/year, and indirect savings from the reduction in complications were $12.86 million/year nationally (Anstey et al., 2019). Masood 2018 clarified the pharmacist-led interventions could reduce the cost of medications for inappropriate SUP pharmacotherapy during the study period from $2,433.00 to $239.80 (Masood et al. (2018)). Buckley 2015 and Coursol 2005 identified the cost of the drugs for SUP per patient and clarified that the pharmacist-led intervention reduced it from $30.52±51.45 to $8.91±11.03 and $8.74 to $6.68 (Coursol and Sanzari, 2005; Buckley et al., 2015).

**TABLE 7:** Economical outcomes related to SUP.

Study ID	Outcome	Pre-	Post-	Other
Anstey et al. (2019)	Direct savings to all Australian intensive care units	—	—	$1.61 million/year
	Indirect savings from the reduction in complications to all Australian intensive care units	—	—	$12.86 million/year
Masood et al. (2018)	Cost of drugs for inappropriate SUP during study period	$2,433.00	$239.80	—
Buckley et al. (2015)	Cost of drugs for SUP per patient	$30.52±51.45	$8.91±11.03	—
Coursol and Sanzari (2005)	Cost of drugs for SUP per patient	$8.74	$6.68	—

## Discussion

### Summary of Evidence

This study was a systematic review of pharmacist-led interventions on the inappropriate use of SUP pharmacotherapy in intensive care units. Although the meta-analysis was not appliable for this review as the heterogeneous of judgment standards for the inappropriate use, we could speculate on the impact of pharmacist-led intervention through narrative synthesis. During hospitalization (7 related studies), the majority (71.4%, 5/7) indicated that pharmacist-led interventions were associated with a decrease in inappropriate SUP pharmacotherapy rates (Coursol and Sanzari, 2005; Tasaka et al., 2014; Buckley et al., 2015; Hammond et al., 2017; Masood et al., 2018). This ratio was 50% (4 related studies) at ICU transfer (Hatch et al., 2010; Buckley et al., 2015) and 60% (5 related studies) at hospital discharged (Hatch et al., 2010; Buckley et al., 2015; Anstey et al., 2019). No studies (4 related studies) found an increased risk of complications related to SUP pharmacotherapy (Coursol and Sanzari, 2005; Buckley et al., 2015; Hammond et al., 2017; Anstey et al., 2019). All studies (100%, 4 related studies) indicated that pharmacist-led intervention was associated with significant costs-savings (Coursol and Sanzari, 2005; Buckley et al., 2015; Masood et al., 2018; Anstey et al., 2019).

Although several SUP guidelines had been published (Armstrong et al., 1999; Vanderbilt University Medical Center, 2005; Guillamondegui et al., 2008; OrlandoRegionalMedicalCenter, 2011; Madsen et al., 2014; Ye et al., 2020), many answers to SUP questions still remain nebulous and need clarification, such as what the relevant anticipated and unanticipated adverse effects of SUP pharmacotherapy are, duration of therapy, and is there a target gastric pH goal for SUP*, etc*. Due to the different implementation time, the indication of SUP pharmacotherapy of the included studies was quite different based on the latest evidence at that time. This also increased the heterogeneity between the included studies. On January 06, 2020, the BMJRapid Recommendation published a new guideline on SUP in ICU patients (Ye et al., 2020). The guideline grouped patients into four categories according to the risk of clinically important GIB and suggested using acid suppression prophylaxis for people with higher risk (4% or higher) and for patients near this threshold, individual values and preferences become more important (Ye et al., 2020). There is currently no studies based on this latest guideline.

Pharmacist interventions varied among the identified studies and included several cointerventions. In general, for identified studies, the pharmacist-led interventions included clarifying indications for SUP pharmacotherapy, education and awareness campaign, review of patients on SUP during rounds and adjustments of drug use. A key role for health-system pharmacists is in the development and implementation of protocols, guidelines, and formularies for directing safe and effective use of medications that focus on patient safety and improved healthcare outcomes (Albanese et al., 2010). In the case of conflicting recommendations in the existing guidelines, only 4 identified studies (50%) had formulated the institution’s protocol. Furthermore, even after the pharmacist’s interventions, the rate of inappropriate use of SUP pharmacotherapy was still high at ICU transfer (3.57–53.39%), which suggests that pharmacists in future studies and clinical practice should focus on the discontinuation of SUP pharmacotherapy. Targeting specific diseases, the pharmacists could stratify patients based on the risk of clinically important GIB and implement different interventions, rather than regarded critically ill patients as a broad target group.

One proposed benefit of pharmacist-led intervention for use of SUP pharmacotherapy is decreased medical expenses. Only 4 studies reported the economic benefits of pharmacist-led interventions improving SUP pharmacotherapy and there was no cost-effectiveness analysis. Further research is needed with economic impact and cost-effectiveness analysis of pharmacist-led intervention.

Only one study was deemed to be of high quality, and most of studies (87.5%) have selection bias, including representativeness of the exposed cohort (87.5%) and ascertainment of exposure (100%). All studies only described the content and deliverer of intervention, but no process outcome being reported, such as the number of a modification proposal made, and the number of suggestions adopted by physicians. In addition, no studies have considered the cost of pharmacist intervention, which is not conducive to stakeholder’s decision-making. Since almost all studies were single center with poorly representative of the community, the conclusions may not extrapolate to other institutions or country.

### Strength and Limitations

Compared with published reviews (Singh-Franco et al., 2020; Orelio et al., 2021), we standardized the calculation process of the inappropriate rate so that the results of the studies were comparable. We also discussed the primary outcome at different time points including during ICU hospitalization, at ICU transfer and hospital discharge. In addition, we fully discussed the heterogeneity between the studies, and have a more correct explanation of the synthesis of the evidence in this review.

Due to the heterogeneity of identified studies, not only the studie’s results, but also the design of studies including the definition of “inappropriate”, the pharmacist’s interventions, and the time of the judgment of appropriateness, it was difficult to precisely identify the impact of pharmacist-led interventions on the inappropriate use of SUP pharmacotherapy in intensive care units and which intervention was more efficient. We excluded several studies because of lacking key data. We were unable to contact the original author for more detailed information, which adds to the bias of this review. Besides, during the recalculation, the rate of inappropriate use of SUP pharmacotherapy at ICU transfer and hospital discharge may be underestimated as we used the SUP pharmacotherapy population during ICU hospitalization as the denominator.

### Implication for Future Study and Practice

This study summarized the current evidence on pharmacist’s role on the management of stress ulcer prophylaxis pharmacotherapy in intensive care units and pointed out the deficiencies in study design that need to be addressed in future studies, thereby contributing to clinical practice. The primary stakeholders of this study are consumers, healthcare professionals, local administrators, national policy makers and other researchers. At present, the participation of pharmacists in a multidisciplinary team is conducive to improving the effectiveness, safety, and economical outcome of patient treatment. However, for specific problems, pharmacists still need more flexible intervention methods and high-quality research to prove the cost-effectiveness of pharmacist interventions. Our research provides reference for pharmacists to participate in SUP drug management and provides methodological reference for future studies.

## Conclusion

Pharmacist-led intervention is associated with a decrease in inappropriate use of SUP pharmacotherapy during hospitalization, at ICU transferred and hospital discharged, and a lot of medical cost savings. Further research is needed to determine whether pharmacist-led intervention is cost-effective.

## Data Availability

The original contributions presented in the study are included in the article/Supplementary Material, further inquiries can be directed to the corresponding author.
