# Supplementary material for: Pharmacist‐Led Intervention on the Inappropriate Use of Stress Ulcer Prophylaxis Pharmacotherapy in Intensive Care Units: A Systematic review
Source: Front Pharmacol. 2021 Oct 25;12:741724. doi: 10.3389/fphar.2021.741724 (PMC8573417; doi:10.3389/fphar.2021.741724)
Supplement: Supplementary file 1 [file DataSheet1.docx]

Supplementary Material

# Supplementary Figures and Tables

## Supplementary tables

**Supplementary Table 1. Search strategy**

| **Chinese Biomedical Literature (Chinese)** |
| --- |
| 1 应激性溃疡 OR 应激性出血 OR 应激性粘膜病变 OR 急性胃粘膜病变 OR Cushing溃疡 OR Curling溃疡 OR 抑酸药 OR H2受体阻滞剂 OR H2受体拮抗剂 OR H2受体阻断药 OR H2受体拮抗药 OR 阻胺H2受体拮抗剂 OR 组胺H2受体阻滞药 OR 阻胺H2拮抗剂 OR尼扎替丁 OR 法莫替丁 OR 西咪替丁 OR 雷尼替丁 OR 硫糖铝 OR 质子泵抑制剂 OR 质子泵抑制药 OR 泮托拉唑 OR 奥美拉唑 OR 雷贝拉唑 OR 埃索美拉唑 OR 兰索拉唑 OR 阻胺H2受体拮抗剂 OR 组胺H2受体阻滞药 OR 阻胺H2拮抗剂  2 药师 OR 药学服务 OR 临床药师 OR 药剂师 OR 药学监护 OR 药学实践  3 重症监护 OR 重症监护室 OR 重症监护病房 OR 监护病房OR ICU OR危重症OR重症  #1 AND #2 AND #3 |
| **Cochrane Central Register of Controlled Trials (English)** |
| ID Search  #1 stress ulcer  #2 Stress Ulceration  #3 Stressful ulcer  #4 irritable ulcer  #5 Acute gastric mucosal lesion  #6 stress ulcer bleeding  #7 cushing ulcer  #8 curling ulcer  #9 stress related mucosal disease  #10 stress related gastrointestinal bleeding  #11 acid-inhibitory drug*  #12 acid inhibitor*  #13 acid-suppressive drug*  #14 sucralfate  #15 burimamide  #16 metiamide  #17 h2ra  #18 h2rb  #19 h2ras  #20 h2rbs  #21 histamine 2 receptor antagonist*  #22 histamine-2 receptor blocker  #23 nizatidine  #24 famotidine  #25 cimetidine  #26 ranitidine  #27 histamine h2 antagonist*  #28 Histamine H2 Receptor Blockader*  #29 Histamine H2 Blocker* 200  #30 Histamine H2 Receptor Antagonist*  #31 H2 Receptor Blockader*  #32 H2 Antihistaminics  #33 ppi  #34 ppis  #35 proton pump inhibitor*  #36 pantoprazole  #37 omeprazole  #38 rabeprazole  #39 dexlansoprazole  #40 esomeprazole  #41 lansoprazole  #42 #1 OR #2 OR #3 OR #4 OR #5 OR #6 OR #7 OR #8 OR #9 OR #10 OR #11 OR #12 OR #13 OR #14 OR #15 OR #16 OR #17 OR #18 OR #19 OR #20 OR #21 OR #22 OR #23 OR #24 OR #25 OR #26 OR #27 OR #28 OR #29 OR #30 OR #31 OR #32 OR #33 OR #34 OR #35 OR #36 OR #37 OR #38 OR #39 OR #40 OR #41  #43 Pharmacist*  #44 Pharmaceutical service*  #45 Pharmaceutical care*  #46 practice-based pharmacist*  #47 clinical pharmacist*  #48 GP* pharmacist*  #49 practice Pharmacist*  #50 general practitioner pharmacist*  #51 Family pharmacist*  #52 Family medicine pharmacist*  #53 Practice pharmacist*  #54 independen* pharmacist*  #55 prescrib* pharmacist*  #56 #43 OR #44 OR #45 OR #46 OR #47 OR #48 OR #49 OR #50 OR #51 OR #52 OR #53 OR #54 OR #55  #57 icu  #58 coronary care  #59 recovery room  #60 par  #61 burn unit  #62 critically ill  #63 cardiac care  #64 intensive care unit  #65 intensive care  #66 critical care  #67 critical illness  #68 coronary care unit  #69 postoperative care  #70 #57 OR #58 OR #59 OR #60 OR #61 OR #62 OR #63 OR #64 OR #65 OR #66 OR #67 OR #68 OR #69  #71 #42 AND #56 AND #70 |
| **the Cochrane Library (English)** |
| ID Search  #1 stress ulcer  #2 Stress Ulceration  #3 Stressful ulcer  #4 irritable ulcer  #5 Acute gastric mucosal lesion  #6 stress ulcer bleeding  #7 cushing ulcer  #8 curling ulcer  #9 stress related mucosal disease  #10 stress related gastrointestinal bleeding  #11 acid-inhibitory drug*  #12 acid inhibitor*  #13 acid-suppressive drug*  #14 sucralfate  #15 burimamide  #16 metiamide  #17 h2ra  #18 h2rb  #19 h2ras  #20 h2rbs  #21 histamine 2 receptor antagonist*  #22 histamine-2 receptor blocker  #23 nizatidine  #24 famotidine  #25 cimetidine  #26 ranitidine  #27 histamine h2 antagonist*  #28 Histamine H2 Receptor Blockader*  #29 Histamine H2 Blocker* 200  #30 Histamine H2 Receptor Antagonist*  #31 H2 Receptor Blockader*  #32 H2 Antihistaminics  #33 ppi  #34 ppis  #35 proton pump inhibitor*  #36 pantoprazole  #37 omeprazole  #38 rabeprazole  #39 dexlansoprazole  #40 esomeprazole  #41 lansoprazole  #42 #1 OR #2 OR #3 OR #4 OR #5 OR #6 OR #7 OR #8 OR #9 OR #10 OR #11 OR #12 OR #13 OR #14 OR #15 OR #16 OR #17 OR #18 OR #19 OR #20 OR #21 OR #22 OR #23 OR #24 OR #25 OR #26 OR #27 OR #28 OR #29 OR #30 OR #31 OR #32 OR #33 OR #34 OR #35 OR #36 OR #37 OR #38 OR #39 OR #40 OR #41  #43 Pharmacist*  #44 Pharmaceutical service*  #45 Pharmaceutical care*  #46 practice-based pharmacist*  #47 clinical pharmacist*  #48 GP* pharmacist*  #49 practice Pharmacist*  #50 general practitioner pharmacist*  #51 Family pharmacist*  #52 Family medicine pharmacist*  #53 Practice pharmacist*  #54 independen* pharmacist*  #55 prescrib* pharmacist*  #56 #43 OR #44 OR #45 OR #46 OR #47 OR #48 OR #49 OR #50 OR #51 OR #52 OR #53 OR #54 OR #55  #57 icu  #58 coronary care  #59 recovery room  #60 par  #61 burn unit  #62 critically ill  #63 cardiac care  #64 intensive care unit  #65 intensive care  #66 critical care  #67 critical illness  #68 coronary care unit  #69 postoperative care  #70 #57 OR #58 OR #59 OR #60 OR #61 OR #62 OR #63 OR #64 OR #65 OR #66 OR #67 OR #68 OR #69  #71 #42 AND #56 AND #70 |
| **China National Knowledge Infrastructure (Chinese)** |
| SU=(应激性溃疡 + 应激性出血 + 应激性粘膜病变 + 急性胃粘膜病变 + Cushing溃疡 + Curling溃疡 + 抑酸药 + H2受体阻滞剂 + H2受体拮抗剂 + H2受体阻断药 + H2受体拮抗药 + 阻胺H2受体拮抗剂 + 组胺H2受体阻滞药 + 阻胺H2拮抗剂 + 尼扎替丁 + 法莫替丁 + 西咪替丁 + 雷尼替丁 + 硫糖铝 + 质子泵抑制剂 + 质子泵抑制药 + 泮托拉唑 + 奥美拉唑 + 雷贝拉唑 + 埃索美拉唑 + 兰索拉唑) AND SU=(药师 + 药学服务 + 临床药师 + 药剂师 + 药学监护 + 药学实践) AND SU=(重症监护 + 重症监护室 + 重症监护病房 + 监护病房 + ICU + 危重症 + 重症) |
| **EMBASE (Ovid, English)** |
| 1 stress ulcer.mp. or exp stress ulcer/  2 exp stress ulcer/ or Stress Ulceration.mp.  3 Stressful ulcer.mp.  4 irritable ulcer.mp.  5 exp stomach mucosa lesion/ or Acute gastric mucosal lesion.mp.  6 stress ulcer bleeding.mp.  7 cushing ulcer.mp.  8 curling ulcer.mp.  9 stress related mucosal disease.mp.  10 stress related gastrointestinal bleeding.mp. or exp gastrointestinal hemorrhage/  11 Anti-Ulcer Agents.mp. or exp antiulcer agent/  12 acid-inhibitory drug.mp.  13 acid inhibitor.mp.  14 acid-suppressive drug.mp.  15 sucralfate.mp. or exp sucralfate/  16 burimamide.mp. or exp burimamide/  17 metiamide.mp. or exp metiamide/  18 exp histamine H2 receptor antagonist/ or h2ra.mp.  19 h2rb.mp.  20 histamine 2 receptor antagonist.mp. or exp histamine H2 receptor antagonist/  21 histamine-2 receptor blocker.mp. or exp histamine H2 receptor antagonist/  22 nizatidine.mp. or exp nizatidine/  23 exp famotidine/ or famotidine.mp.  24 cimetidine.mp. or exp cimetidine/  25 exp ranitidine/ or ranitidine.mp.  26 histamine h2 antagonist.mp.  27 Histamine H2 Receptor Blockader.mp.  28 Histamine H2 Blocker.mp.  29 Histamine H2 Receptor Antagonist.mp.  30 H2 Receptor Blockader.mp.  31 H2 Antihistaminics.mp.  32 ppi.mp. or exp proton pump inhibitor/  33 proton pump inhibitor.mp.  34 pantoprazole.mp. or exp pantoprazole/  35 omeprazole.mp. or exp omeprazole/  36 rabeprazole.mp. or exp rabeprazole/  37 dexlansoprazole.mp. or exp dexlansoprazole/  38 exp esomeprazole/ or esomeprazole.mp.  39 exp lansoprazole/ or lansoprazole.mp.  40 1 or 2 or 3 or 4 or 5 or 6 or 7 or 8 or 9 or 10 or 11 or 12 or 13 or 14 or 15 or 16 or 17 or 18 or 19 or 20 or 21 or 22 or 23 or 24 or 25 or 26 or 27 or 28 or 29 or 30 or 31 or 32 or 33 or 34 or 35 or 36 or 37 or 38 or 39  41 Pharmacist.mp. or exp pharmacist/  42 Pharmaceutical service.mp.  43 Pharmaceutical care.mp. or exp pharmaceutical care/  44 practice-based pharmacist.mp.  45 clinical pharmacist.mp. or exp clinical pharmacist/  46 GP* pharmacist.mp.  47 practice Pharmacist.mp.  48 general practitioner pharmacist.mp.  49 Family pharmacist.mp.  50 Family medicine pharmacist.mp.  51 independen* pharmacist.mp.  52 prescrib* pharmacist.mp.  53 41 or 42 or 43 or 44 or 45 or 46 or 47 or 48 or 49 or 50 or 51 or 52  54 icu.mp. or exp intensive care unit/  55 exp coronary care unit/ or coronary care.mp.  56 recovery room.mp. or exp recovery room/  57 par.mp.  58 burn unit.mp. or exp burn unit/  59 critically ill.mp. or exp critically ill patient/  60 cardiac care.mp.  61 intensive care unit.mp.  62 intensive care.mp. or exp intensive care/  63 critical care.mp.  64 critical illness.mp. or exp critical illness/  65 coronary care unit.mp.  66 postoperative care.mp. or exp postoperative care/  67 54 or 55 or 56 or 57 or 58 or 59 or 60 or 61 or 62 or 63 or 64 or 65 or 66  68 40 and 53 and 67 |
| **PubMed (English)** |
| ((((((((((((((((((((((((((burimamide[Text Word] OR metiamide[Text Word] OR h2ra[Text Word] OR h2rb[Text Word] OR h2ras[Text Word] OR h2rbs[Text Word] OR histamine 2 receptor antagonist*[Text Word] OR histamine-2 receptor blocker[Text Word] OR nizatidine[Text Word] OR famotidine[Text Word] OR cimetidine[Text Word] OR ranitidine[Text Word] OR histamine h2 antagonist*[Text Word] OR Histamine H2 Receptor Blockader*[Text Word] OR Histamine H2 Blocker*[Text Word] OR Histamine H2 Receptor Antagonist*[Text Word] OR H2 Receptor Blockader*[Text Word] OR H2 Antihistaminics[Text Word])) OR (ppi[Text Word] OR ppis[Text Word] OR proton pump inhibitor*[Text Word] OR pantoprazole[Text Word] OR omeprazole[Text Word] OR rabeprazole[Text Word] OR dexlansoprazole[Text Word] OR esomeprazole[Text Word] OR lansoprazole[Text Word])) OR sucralfate[Text Word])) OR ((acid-inhibitory drug* OR acid inhibitor* OR acid-suppressive drug* OR Anti-Ulcer Agents))) OR ("Anti-Ulcer Agents" [Pharmacological Action] OR "Anti-Ulcer Agents")) OR "Burimamide") OR "Metiamide") OR ("Histamine H2 Antagonists" AND "Histamine H2 Antagonists" [Pharmacological Action])) OR ("Nizatidine" OR "nizatidine sulfoxide" [Supplementary Concept])) OR "Famotidine") OR "Cimetidine") OR "Ranitidine") OR ("Proton Pump Inhibitors" OR "Proton Pump Inhibitors" [Pharmacological Action])) OR "Pantoprazole") OR ("Omeprazole" OR "Esomeprazole")) OR "Rabeprazole") OR "Dexlansoprazole") OR ("Lansoprazole" OR "Dexlansoprazole")) OR ("Histamine H2 Antagonists" [Pharmacological Action] OR "Histamine H2 Antagonists")) OR "Sucralfate")) OR ((stress ulcer[Text Word] OR Stress Ulceration[Text Word] OR Stressful ulcer[Text Word] OR irritable ulcer[Text Word] OR Acute gastric mucosal lesion[Text Word] OR stress ulcer bleeding[Text Word] OR cushing ulcer[Text Word] OR curling ulcer[Text Word] OR stress related mucosal disease[Text Word] OR stress related gastrointestinal bleeding[Text Word])))) AND (((((((((((icu[Text Word] OR coronary care[Text Word] OR recovery room[Text Word] OR par[Text Word] OR burn unit[Text Word] OR critically ill[Text Word] OR cardiac care[Text Word] OR intensive care unit[Text Word] OR intensive care[Text Word] OR critical care[Text Word] OR critical illness[Text Word] OR coronary care unit[Text Word] OR postoperative care[Text Word]))) OR "Postoperative Care") OR "Coronary Care Units") OR "Critical Illness") OR ("Critical Care" OR "Intensive Care, Neonatal" OR "Intensive Care Units, Pediatric" OR "Intensive Care Units, Neonatal" OR "Intensive Care Units" OR "Critical Care Nursing")) OR "Burn Units") OR "Recovery Room") OR ("Coronary Care Units" OR "Cardiovascular Nursing")) OR ("Intensive Care Units" OR "Intensive Care Units, Neonatal"))) AND (((((Pharmacist*[Text Word] OR Pharmaceutical service*[Text Word] OR Pharmaceutical care*[Text Word] OR practice-based pharmacist*[Text Word] OR clinical pharmacist*[Text Word] OR GP* pharmacist*[Text Word] OR practice Pharmacist*[Text Word] OR general practitioner pharmacist*[Text Word] OR Family pharmacist*[Text Word] OR Family medicine pharmacist*[Text Word] OR Practice pharmacist*[Text Word] OR independen* pharmacist*[Text Word] OR prescrib* pharmacist*[Text Word]))) OR ("Pharmaceutical Services" OR "Pharmaceutical Services, Online" OR "Pharmacy Service, Hospital" OR "Community Pharmacy Services")) OR "Pharmacists") |
| **VIP (Chinese)** |
| M=(应激性溃疡 + 应激性出血 + 应激性粘膜病变 + 急性胃粘膜病变 + Cushing溃疡 + Curling溃疡 + 抑酸药 + H2受体阻滞剂 + H2受体拮抗剂 + H2受体阻断药 + H2受体拮抗药 + 阻胺H2受体拮抗剂 + 组胺H2受体阻滞药 + 阻胺H2拮抗剂 + 尼扎替丁 + 法莫替丁 + 西咪替丁 + 雷尼替丁 + 硫糖铝 + 质子泵抑制剂 + 质子泵抑制药 + 泮托拉唑 + 奥美拉唑 + 雷贝拉唑 + 埃索美拉唑 + 兰索拉唑) AND M=(药师 + 药学服务 + 临床药师 + 药剂师 + 药学监护 + 药学实践) AND M=(重症监护 + 重症监护室 + 重症监护病房 + 监护病房 + ICU + 危重症 + 重症) |
| **Wanfang (Chinese)** |
| 主题:(应激性溃疡 + 应激性出血 + 应激性粘膜病变 + 急性胃粘膜病变 + Cushing溃疡 + Curling溃疡 + 抑酸药 + H2受体阻滞剂 + H2受体拮抗剂 + H2受体阻断药 + H2受体拮抗药 + 阻胺H2受体拮抗剂 + 组胺H2受体阻滞药 + 阻胺H2拮抗剂 + 尼扎替丁 + 法莫替丁 + 西咪替丁 + 雷尼替丁 + 硫糖铝 + 质子泵抑制剂 + 质子泵抑制药 + 泮托拉唑 + 奥美拉唑 + 雷贝拉唑 + 埃索美拉唑 + 兰索拉唑) AND 主题:(药师 + 药学服务 + 临床药师 + 药剂师 + 药学监护 + 药学实践) AND 主题:(重症监护 + 重症监护室 + 重症监护病房 + 监护病房 + ICU + 危重症 + 重症) |

**Supplementary Table 2. Indication for initiation of** **SUP pharmacotherapy ^*△^**

| **Indication** | **Study ID** | | | | | | | **Amount** |
| --- | --- | --- | --- | --- | --- | --- | --- | --- |
|  | **Anstey 2019^[22]^** | **Masood 2018^[23]^** | **Hammond 2017^[24]^** | **Buckley 2015^[14]^** | **Tasaka 2014^[26]^** | **Hatch 2010^[27]^** | **Coursol 2005^[28]^** |  |
| One of the following: | | | | | | | | |
| Mechanical ventilation for >48 hours | ● | ● | ● | ● | ● | ● | ● | 7 |
| Coagulopathy | ● | ● | ● | ● | ● | ● | ● | 7 |
| Severe traumatic brain injury or spinal injury | ● | ● |  |  | ● | ● | ● | 5 |
| prior history of gastric ulceration or bleeding within 1 year | ● | ● |  |  | ● | ● | ● | 5 |
| Severe burns | ● | ● |  |  |  | ● | ● | 4 |
| Multiple trauma |  | ● |  |  | ● | ● | ● | 4 |
| Glasgow coma score ≤10 |  | ● |  |  |  | ● |  | 2 |
| Postop transplant patient (de novo) |  | ● |  |  |  | ● |  | 2 |
| Liver failure or partial hepatectomy |  |  |  |  |  | ● | ● | 2 |
| Renal failure |  | ● |  |  |  |  |  | 1 |
| Hepatic failure |  | ● |  |  |  |  |  | 1 |
| Multiple organ failure ≥3 organs |  |  |  |  |  |  | ● | 1 |
| ICU populations |  | ● |  |  |  |  |  | 1 |
| Two or more of following: | | | | | | | | |
| High-dose glucocorticoid use | ● | ● | ● |  | ● | ● |  | 5 |
| Severe sepsis or septic shock | ● | ● |  |  | ● | ● |  | 4 |
| Postoperative transplantation | ● |  | ● | ● |  |  |  | 3 |
| ICU stay >1 week |  | ● |  |  |  | ● |  | 2 |
| Occult bleeding lasting ＞6 days |  | ● |  |  |  | ● |  | 2 |
| Liver failure with associated coagulopathy | ● |  | ● |  |  |  |  | 2 |
| Renal failure | ● |  |  |  |  |  |  | 1 |
| History of gastrointestinal (GI) ulcer/bleeding within 1 year |  |  | ● |  |  |  |  | 1 |
| Severe burn |  |  | ● |  |  |  |  | 1 |
| Head or spinal cord injury |  |  | ● |  |  |  |  | 1 |
| Acute organ dysfunction (including acute kidney injury) |  |  | ● |  |  |  |  | 1 |
| Hypoperfusion |  |  | ● |  |  |  |  | 1 |
| Major surgery |  |  | ● |  |  |  |  | 1 |
| Multiple trauma |  |  | ● |  |  |  |  | 1 |

* One study (Fan 2015^[25]^) not report the indication for SUP was excluded from this table.

△The definitions of risk factors varied between studies and we incorporated risk factors without considering the difference in definition.

**Supplementary Table 3** **Cessation of SUP**

| **Cessation of SUP** | **Study ID** |
| --- | --- |
| No ongoing indication | Anstey 2019^[22]^, Hammond 2017^[24]^, Buckley 2015^[14]^, Tasaka 2014^[26]^ |
| Discharge from ICU | Masood 2018^[23]^, Tasaka 2014^[26]^ |
| Receiving enteral feeding | Anstey 2019^[22]^ |
| No mention | Fan 2015^[25]^, Hatch 2010^[27]^, Coursol 2005^[28]^ |

# PRISMA checklist

| **Section and Topic** | **Item #** | **Checklist item** | **Location where item is reported** |
| --- | --- | --- | --- |
| **TITLE** | | |  |
| Title | 1 | Identify the report as a systematic review. | Line 106-107 |
| **ABSTRACT** | | |  |
| Abstract | 2 | See the PRISMA 2020 for Abstracts checklist. | Line 31-53 |
| **INTRODUCTION** | | |  |
| Rationale | 3 | Describe the rationale for the review in the context of existing knowledge. | Line 56-101 |
| Objectives | 4 | Provide an explicit statement of the objective(s) or question(s) the review addresses. | Line 102-105 |
| **METHODS** | | |  |
| Eligibility criteria | 5 | Specify the inclusion and exclusion criteria for the review and how studies were grouped for the syntheses. | Line 111-125 |
| Information sources | 6 | Specify all databases, registers, websites, organisations, reference lists and other sources searched or consulted to identify studies. Specify the date when each source was last searched or consulted. | Line 127-141 |
| Search strategy | 7 | Present the full search strategies for all databases, registers and websites, including any filters and limits used. | Line 142-147 |
| Selection process | 8 | Specify the methods used to decide whether a study met the inclusion criteria of the review, including how many reviewers screened each record and each report retrieved, whether they worked independently, and if applicable, details of automation tools used in the process. | Line 148-152 |
| Data collection process | 9 | Specify the methods used to collect data from reports, including how many reviewers collected data from each report, whether they worked independently, any processes for obtaining or confirming data from study investigators, and if applicable, details of automation tools used in the process. | Line 153-156 |
| Data items | 10a | List and define all outcomes for which data were sought. Specify whether all results that were compatible with each outcome domain in each study were sought (e.g. for all measures, time points, analyses), and if not, the methods used to decide which results to collect. | Line 165-166 |
|  | 10b | List and define all other variables for which data were sought (e.g. participant and intervention characteristics, funding sources). Describe any assumptions made about any missing or unclear information. | Line 156-162 |
| Study risk of bias assessment | 11 | Specify the methods used to assess risk of bias in the included studies, including details of the tool(s) used, how many reviewers assessed each study and whether they worked independently, and if applicable, details of automation tools used in the process. | Line 163 |
| Effect measures | 12 | Specify for each outcome the effect measure(s) (e.g. risk ratio, mean difference) used in the synthesis or presentation of results. | Line 201-206 |
| Synthesis methods | 13a | Describe the processes used to decide which studies were eligible for each synthesis (e.g. tabulating the study intervention characteristics and comparing against the planned groups for each synthesis (item #5)). | Line 173-200 |
|  | 13b | Describe any methods required to prepare the data for presentation or synthesis, such as handling of missing summary statistics, or data conversions. | Line 173-200 |
|  | 13c | Describe any methods used to tabulate or visually display results of individual studies and syntheses. | Line 167-172 |
|  | 13d | Describe any methods used to synthesize results and provide a rationale for the choice(s). If meta-analysis was performed, describe the model(s), method(s) to identify the presence and extent of statistical heterogeneity, and software package(s) used. | Line 173-200 |
|  | 13e | Describe any methods used to explore possible causes of heterogeneity among study results (e.g. subgroup analysis, meta-regression). | - |
|  | 13f | Describe any sensitivity analyses conducted to assess robustness of the synthesized results. | - |
| Reporting bias assessment | 14 | Describe any methods used to assess risk of bias due to missing results in a synthesis (arising from reporting biases). | - |
| Certainty assessment | 15 | Describe any methods used to assess certainty (or confidence) in the body of evidence for an outcome. | - |
| **RESULTS** | | |  |
| Study selection | 16a | Describe the results of the search and selection process, from the number of records identified in the search to the number of studies included in the review, ideally using a flow diagram. | Line 208-217 |
|  | 16b | Cite studies that might appear to meet the inclusion criteria, but which were excluded, and explain why they were excluded. | Line 212-213 and figure 2 |
| Study characteristics | 17 | Cite each included study and present its characteristics. | Line 218-226 and table 2 |
| Risk of bias in studies | 18 | Present assessments of risk of bias for each included study. | Line 227-232 |
| Results of individual studies | 19 | For all outcomes, present, for each study: (a) summary statistics for each group (where appropriate) and (b) an effect estimate and its precision (e.g. confidence/credible interval), ideally using structured tables or plots. | Line 250-284 and table 1,5-7 |
| Results of syntheses | 20a | For each synthesis, briefly summarise the characteristics and risk of bias among contributing studies. | Line 250-284 |
|  | 20b | Present results of all statistical syntheses conducted. If meta-analysis was done, present for each the summary estimate and its precision (e.g. confidence/credible interval) and measures of statistical heterogeneity. If comparing groups, describe the direction of the effect. | Line 250-284 |
|  | 20c | Present results of all investigations of possible causes of heterogeneity among study results. | - |
|  | 20d | Present results of all sensitivity analyses conducted to assess the robustness of the synthesized results. | - |
| Reporting biases | 21 | Present assessments of risk of bias due to missing results (arising from reporting biases) for each synthesis assessed. | - |
| Certainty of evidence | 22 | Present assessments of certainty (or confidence) in the body of evidence for each outcome assessed. | - |
| **DISCUSSION** | | |  |
| Discussion | 23a | Provide a general interpretation of the results in the context of other evidence. |  |
|  | 23b | Discuss any limitations of the evidence included in the review. | Line 339-327 |
|  | 23c | Discuss any limitations of the review processes used. | Line 334-338 |
|  | 23d | Discuss implications of the results for practice, policy, and future research. | Line 348-358 |
| **OTHER INFORMATION** | | |  |
| Registration and protocol | 24a | Provide registration information for the review, including register name and registration number, or state that the review was not registered. | Line 109-110 |
|  | 24b | Indicate where the review protocol can be accessed, or state that a protocol was not prepared. | - |
|  | 24c | Describe and explain any amendments to information provided at registration or in the protocol. | - |
| Support | 25 | Describe sources of financial or non-financial support for the review, and the role of the funders or sponsors in the review. | Line 381-383 |
| Competing interests | 26 | Declare any competing interests of review authors. | Line 378-379 |
| Availability of data, code and other materials | 27 | Report which of the following are publicly available and where they can be found: template data collection forms; data extracted from included studies; data used for all analyses; analytic code; any other materials used in the review. | Line 375-376 |

*From:*  Page MJ, McKenzie JE, Bossuyt PM, Boutron I, Hoffmann TC, Mulrow CD, et al. The PRISMA 2020 statement: an updated guideline for reporting systematic reviews. BMJ 2021;372:n71. doi: 10.1136/bmj.n71

For more information, visit: <http://www.prisma-statement.org/>
